# Data-driven interdisciplinary mathematical modelling quantitatively unveils competition dynamics of co-circulating influenza strains

**DOI:** 10.1186/s12967-017-1269-6

**Published:** 2017-07-28

**Authors:** Bin-Shenq Ho, Kun-Mao Chao

**Affiliations:** 1 Department of Computer Science and Information Engineering, National Taiwan University, Taipei, Taiwan, ROC; 2 Public Health Bureau, Hsinchu, Taiwan, ROC; 3 Taiwan Centers for Disease Control, Taipei, Taiwan, ROC; 4 Graduate Institute of Biomedical Electronics and Bioinformatics, National Taiwan University, Taipei, Taiwan, ROC

**Keywords:** Influenza, Transmission, Strain competition, Quantitative modelling

## Abstract

**Background:**

Co-circulation of influenza strains is common to seasonal epidemics and pandemic emergence. Competition was considered involved in the vicissitudes of co-circulating influenza strains but never quantitatively studied at the human population level. The main purpose of the study was to explore the competition dynamics of co-circulating influenza strains in a quantitative way.

**Methods:**

We constructed a heterogeneous dynamic transmission model and ran the model to fit the weekly A/H1N1 influenza virus isolation rate through an influenza season. The construction process started on the 2007–2008 single-clade influenza season and, with the contribution from the clade-based A/H1N1 epidemiological curves, advanced to the 2008–2009 two-clade influenza season. Pearson method was used to estimate the correlation coefficient between the simulated epidemic curve and the observed weekly A/H1N1 influenza virus isolation rate curve.

**Results:**

The model found the potentially best-fit simulation with correlation coefficient up to 96% and all the successful simulations converging to the best-fit. The annual effective reproductive number of each co-circulating influenza strain was estimated. We found that, during the 2008–2009 influenza season, the annual effective reproductive number of the succeeding A/H1N1 clade 2B-2, carrying H275Y mutation in the neuraminidase, was estimated around 1.65. As to the preceding A/H1N1 clade 2C-2, the annual effective reproductive number would originally be equivalent to 1.65 but finally took on around 0.75 after the emergence of clade 2B-2. The model reported that clade 2B-2 outcompeted for the 2008–2009 influenza season mainly because clade 2C-2 suffered from a reduction of transmission fitness of around 71% on encountering the former.

**Conclusions:**

We conclude that interdisciplinary data-driven mathematical modelling could bring to light the transmission dynamics of the A/H1N1 H275Y strains during the 2007–2009 influenza seasons worldwide and may inspire us to tackle the continually emerging drug-resistant A/H1N1pdm09 strains. Furthermore, we provide a prospective approach through mathematical modelling to solving a seemingly unintelligible problem at the human population level and look forward to its application at molecular level through bridging the resolution capacities of related disciplines.

**Electronic supplementary material:**

The online version of this article (doi:10.1186/s12967-017-1269-6) contains supplementary material, which is available to authorized users.

## Background

Over the past 100 years, the three major influenza pandemics, namely the 1918 pandemic, 1957 pandemic, and 1968 pandemic, claimed millions of human lives [1, 2]. A number of authors used simulation models to explore the optimal strategies of containing a potential influenza threat [3–8]. The results not only provided insights into the control strategies of influenza pandemics but also shed light on the resolution of some enigmas of the seasonal influenza epidemics. One common interest pertinent to seasonal epidemics and pandemic emergence concerns the competition dynamics of co-circulating strains.

Given the ever worldwide dissemination of drug-resistant seasonal A/H1N1 H275Y variants during the 2007–2009 influenza seasons, the detection of drug-resistant A/H1N1pdm09 variants was of growing concern [9–17]. On one hand, surveillance systems tried to track and analyze the evolution and epidemiology of influenza viruses for better vaccine strain selection and timely antiviral susceptibility assessment [18]. On the other hand, nevertheless, the transmission dynamics of drug-resistant seasonal A/H1N1 H275Y variants was still mysterious at the human population level.

According to Yang et al. [19], A/H1N1 H275Y variants were seldom isolated in Taiwan until September 2008 when they were found emerging in 14.3% of A/H1N1 isolates. As A/H1N1 subtype progressively prevailed, the proportion of A/H1N1 H275Y strains ultimately reached 100% by the end of year 2008. The vast majority of A/H1N1 H275Y strains belonged to clade 2B-2, which dominated the 2008–2009 influenza season in Taiwan. Worthy of note, however, were the preceding strains belonging to A/H1N1 clade 2C-2, which emerged in June 2008 but disappeared before the peak of the 2008–2009 influenza season.

We therefore constructed a transmission model to simulate the competition dynamics of co-circulating clades 2C-2 and 2B-2 during the 2008–2009 influenza season in Taiwan. Virological factors, epidemiological observations, social factors, and climatic factors were incorporated into the model. Annual influenza vaccination program was set accordingly to simulate the real situation. As to antivirals, oseltamivir was the major antiviral stockpile then but was documented as rarely prescribed before May 2009 in Taiwan [19, 20]. Taking the advantage of the antiviral-free scenario, we studied the competition dynamics between the preceding clade 2C-2 and the succeeding clade 2B-2 in a quantitative way.

## Methods

### Virological data

We intended to develop a heterogeneous dynamic transmission model and ran the model to fit the weekly A/H1N1 influenza virus isolation rate through an influenza season. We inspected the laboratory data of the national influenza surveillance system coordinated by Taiwan Centers for Disease Control from week 1 (starting on January 1) of 2007 through week 26 (ending on June 28) of 2009. The surveillance system covered approximately 75% of the 352 basic administrative units throughout the northern, central, southern, and eastern regions of Taiwan [19, 21, 22]. Considering the inherent variability of the weekly data, we adopted the aggregate weekly A/H1N1 influenza virus isolation rate (IVIR) of each week, computed from all positive isolations divided by all collected specimens of the three consecutive weeks, for the model to simulate (see Additional file 1: Table S1). To accommodate to the time frame of the model, i.e., 30 days a month and 12 months a year, we further reframed the time frame of the aggregate weekly data so that 52 weeks a year were transformed into 360 days a year. April 1 was arbitrarily defined as day 1 of the model.

### Epidemiological evidence

As shown in Yang et al. [19], the 2007–2008 seasonal influenza epidemic, dominated by A/H1N1 clade 2B-1, peaked in January of 2008, and the 2008–2009 seasonal influenza epidemic, dominated by clade 2B-2, peaked in February of 2009. Besides, an earlier hillock of clade 2C-2 occurred during the period from September through October of 2008. To simulate the weekly A/H1N1 IVIR curve, the model hereby assumed the peaks of the 2007–2008 and 2008–2009 seasonal influenza epidemics were day 267 and day 304, respectively. For the 2008–2009 epidemic, as evidenced in Yang et al. [19], the model further assumed day 180 as the hillock peak, with a height between 7/192 and 10/192 the height of the epidemic peak, and assumed the crossover occurred on day 210.

### Demographic data and age-specific contact rates

The model population adopted the mid-year population structure of the year, 2007 and 2008 respectively, and was classified into six age groups: 0–5, 6–12, 13–19, 20–39, 40–59, and ≥60 years (see Additional file 2: Table S2). The mid-year age-structured population sizes were obtained from Department of Household Registration, Ministry of the Interior (http://www.ris.gov.tw/zh_TW/346). Births and deaths were neglected because of the relatively short time scale that the simulation spanned. The model further adopted the normalized age-specific contact rates estimated in Wallinga et al. [23]. See Additional file 3: Appendix, for details.

### Seasonality modulated by absolute humidity

According to Shaman et al. [24, 25], under the assumption of influenza transmission seasonality being driven by absolute humidity, the following equation was adopted to modulate influenza transmissibility in the model:

1 $$R_{\text{e}} = R_{{ 0 {\text{min}}}} + e^{{\left( { - 1 8 0\,\times\,{\text{SH}}\,+\,{\text{log }}\left( {R_{{ 0 {\text{max}}}}\,-\,R_{{ 0 {\text{min}}}} } \right)} \right)}}$$

where *R*_e_, effective reproductive number, defined as the actual average number of secondary cases per primary case during a time period, was adopted to estimate the transmission rate coefficient during the period. *R*_0max_ and *R*_0min_ represented the maximum and minimum daily basic reproductive numbers, respectively, and *SH* represented specific humidity and acted as proxy of absolute humidity. The meteorological variables used to derive specific humidity could be referred to Taiwan Central Weather Bureau (http://www.cwb.gov.tw/V7/climate/monthlyData/mD.htm). The relevant data from April 2007 through June 2009 were shown in Additional file 4: Table S3. See Additional file 3: Appendix, for details.

### Transmission model construction

We proceeded to construct a transmission model with four dimensions taken into consideration, i.e., virological factors, epidemiological observations, social factors, and climatic factors [26]. We started on constructing an susceptible-exposed-infective-recovered (*SEIR*) compartmental model as the backbone [27]. The equations below described the deterministic behaviors of the model:

2 $$\frac{{{\text{d}}S\left[ i \right]\left( t \right)}}{{{\text{d}}t}} = - \lambda \left[ i \right]S\left[ i \right]\left( t \right)$$

3 $$\frac{{{\text{d}}E\left[ i \right]\left( t \right)}}{{{\text{d}}t}} = \lambda \left[ i \right]S\left[ i \right]\left( t \right) - \theta E\left[ i \right]\left( t \right)$$

4 $$\frac{{{\text{d}}I\left[ i \right]\left( t \right)}}{{{\text{d}}t}} = \theta E\left[ i \right]\left( t \right) - \alpha I\left[ i \right]\left( t \right)$$

5 $$\frac{{{\text{d}}R\left[ i \right]\left( t \right)}}{{{\text{d}}t}} = \alpha I\left[ i \right]\left( t \right)$$

where *S*[*i*], *E*[*i*], *I*[*i*], and *R*[*i*] represented the size of age group *i* in compartment *S*, *E*, *I*, and *R*, respectively (see Table 1 for details of the parameters). The relationship between *R*_e_ and the λ[*i*] and the interaction between various age groups were illustrated in Appendix (see Additional file 3).

**Table 1:** Summary of model parameter values

Parameter	Meaning	Value	Source/comment
*R*_0max_, *R*_0min_	Defining the range of the basic reproductive number	See Ref. [25]	The simulations were performed with the parameters *R*_0max_ set ranging from 2.40 to 1.20 and *R*_0min_ set ranging from *R*_0max_ to 0.80
*Temp*	Mean temperature	See Additional file 4: Table S3	Taiwan Central Weather Bureau (http://www.cwb.gov.tw/V7/climate/monthlyData/mD.htm)
*RH*	Mean relative humidity	See Additional file 4: Table S3	Taiwan Central Weather Bureau (http://www.cwb.gov.tw/V7/climate/monthlyData/mD.htm)
*AP*	Atmospheric pressure	See Additional file 4: Table S3	Taiwan Central Weather Bureau (http://www.cwb.gov.tw/V7/climate/monthlyData/mD.htm)
*RF*	Relative fitness	Estimated	In terms of transmissibility, representing the strain fitness of a clade as compared with the reference clade
*CC*	Competition cost	Estimated	In terms of transmissibility, addressing the reduction in strain fitness associated with the interaction between two clades
*λ*[*i*]	Force of infection to age group *i*	Estimated	
*θ*	Transition rate from exposed state to infective state	1/day	Probability of the exposed to become infective per unit of time; assuming latent period around 1 day [26]
*α*	Recovery rate	0.2/day	Probability of the infectives to become recovered and immune per unit of time; assuming infectious period around 5 days [26]
*ν*	Proportion of susceptibles becoming immune 15 days after vaccination	See Additional file 5: Table S4, for the progress of annual vaccination program	As the simulated clades were predominantly reported as low reactors to the contemporaneous annual vaccine strains, the model assumed 50% of the vaccinees were protected from infection of the contemporaneous seasonal strains [28, 29]. The assumption was relaxed in the process of assessing the impacts of the annual vaccination program on the 2008–2009 seasonal influenza epidemic (see the “Results” section)

The criteria of a successful simulation were defined correspondingly for single-clade epidemic simulation of the 2007–2008 influenza season and for two-clade epidemic simulation of the 2008–2009 influenza season (see the “Results” section for details). The simulation spanned 15 months, covering the length of an annual influenza season and extending 3 months forward so that the observed emerging stage of the seasonal influenza epidemic could be included (see Additional file 3: Appendix, for details).

However, viewing the exploration that it would be impossible to reproduce the scenario of the 2008–2009 influenza season unless the preceding clade incurred reduction in transmissibility on encountering the succeeding clade, the model further tried probing into strain fitness in two ways. On one hand, since we were interested in whether the succeeding clade per se had fitness advantage over the preceding one, we defined relative fitness (*RF*) to quantify the ratio of transmissibility, in terms of *R*_e_, of the former over the latter as the expression below.

6 $$RF = R_{{{\text{e}}\;{\text{succeeding}}\;{\text{clade}}}} /R_{{{\text{e}}\;{\text{preceding}}\;{\text{clade}}}}$$

On the other hand, because we were also interested in whether the preceding clade suffered from transmissibility impairment when encountering the succeeding one, we defined competition cost (*CC*) to quantify the possible reduction in transmissibility of the former associated with the interaction between the two clades as the expression below.

7 $$CC = 1{ - }(R_{{{\text{e}}\;{\text{preceding}}\;{\text{clade/succeeding}}\;{\text{clade}}}} /R_{{{\text{e}}\;{\text{preceding}}\;{\text{clade}}}} )$$

### Vaccination against circulating strains

In our model, clade *X*, *Y*, and *Z* represented A/H1N1 clade 2B-1, 2C-2, and 2B-2, respectively. All three clades were predominantly reported as low reactors to the contemporaneous annual vaccine strains [30]. We therefore assumed 50% of the vaccinees were protected from infection of the contemporaneous seasonal strains [28, 29] and acquired full immunity 15 days after vaccination [31]. For simplicity, full cross-immunity was further assumed for clades *Y* and *Z*, and people infected by either clade entered into compartment *R* without differentiation. We assumed that all those receiving vaccination were originally in compartment *S* rather than compartment *E*, *I*, or *R*. Hence, our model moved the vaccinated of each age group in the compartment *S*, adjusted by vaccine efficacy, directly into compartment *R* on a daily basis 15 days after vaccination [32, 33]. See Additional file 5: Table S4, for the progress of the annual influenza vaccination program.

### Statistical analysis

The size of compartment *I*, under the assumption that the infectives could reflect the influenza virus isolation rate, was set to track the virological data as well as epidemiological evidence in pursuit of high correlation. Pearson method was used to estimate the correlation coefficient between the simulated epidemic curve and the observed weekly A/H1N1 influenza virus isolation rate curve.

### Programming language and software environment

All mathematical modelling, simulations, calculations, graphical outputs and statistical analyses were performed with R version 3.0.1(https://www.r-project.org).

## Results

### Model

**Fig. 1:**
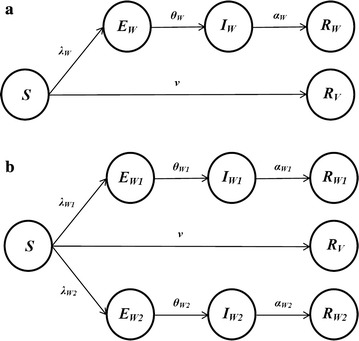
The *SEIR* model. Individuals in the susceptible compartment (*S*) progress along the exposed compartment (*E*), infective compartment (*I*), and recovered compartment (*R*) at per capita rates *λ*, *θ*, and *α*, respectively, once they are infected with influenza. The subscript, *W*, *W1*, *W2*, and *V*, specifies the transition process involving each respective clade/strain. Individuals pass from compartment *S* directly into compartment *R* 15 days after receiving influenza vaccination at a proportion of *ν* according to the program agenda (see Additional file 5: Table S4). **a** Model for single-clade influenza season. **b** Model for two-clade influenza season. The model applied to each age group as well as the whole population

We constructed a heterogeneous dynamic transmission model [27]. The core of the model is schematically represented in Fig. 1.

### Fitting single-clade influenza season and estimating effective reproductive numbers

We first ran the model under the context of the 2007–2008 influenza season and introduced clade *X* on April 1, 2007. A successful simulation was defined as follows: (a) the correlation coefficient of at least 95% to the observed weekly A/H1N1 IVIR curve and (b) the epidemic peak at most 3 days away from the one of the weekly A/H1N1 IVIR curve (day 267) (see “Methods” for details). Through fitting the simulation to the weekly A/H1N1 IVIR curve, the model found not only successful simulations but also the potentially best one. The ten best-fit simulations, ranked by the correlation coefficients, were shown in Table 2 (see Table 1 for details of the parameters). For the first parameter set, the model generated an epidemic curve of clade *X* that peaked on day 269 and was positively correlated to the observed weekly A/H1N1 IVIR curve with the correlation coefficient up to 96.83%. The monthly *R*_e_’s of the 15 months spanned by the simulation, corresponding to the months from April 2007 through June 2008, were estimated around 1.33, 1.32, 1.31, 1.31, 1.31, 1.31, 1.32, 1.33, 1.34, 1.35, 1.36, 1.34, 1.32, 1.32, and 1.31 (see Additional file 6: Table S5). The annual *R*_e_ of clade *X* was hereby estimated around 1.32. The model therefore could successfully identify the best combination of *R*_0max_ and *R*_0min_ and evaluate the *R*_e_ of the single-clade influenza season. In other words, in terms of the *R*_e_ estimated, the first parameter set along with the actual data embedded in the model could establish an epidemic 96.83% correlated to the real epidemic of the 2007–2008 influenza season dominated by A/H1N1 clade 2B-1.

**Fig. 2:**
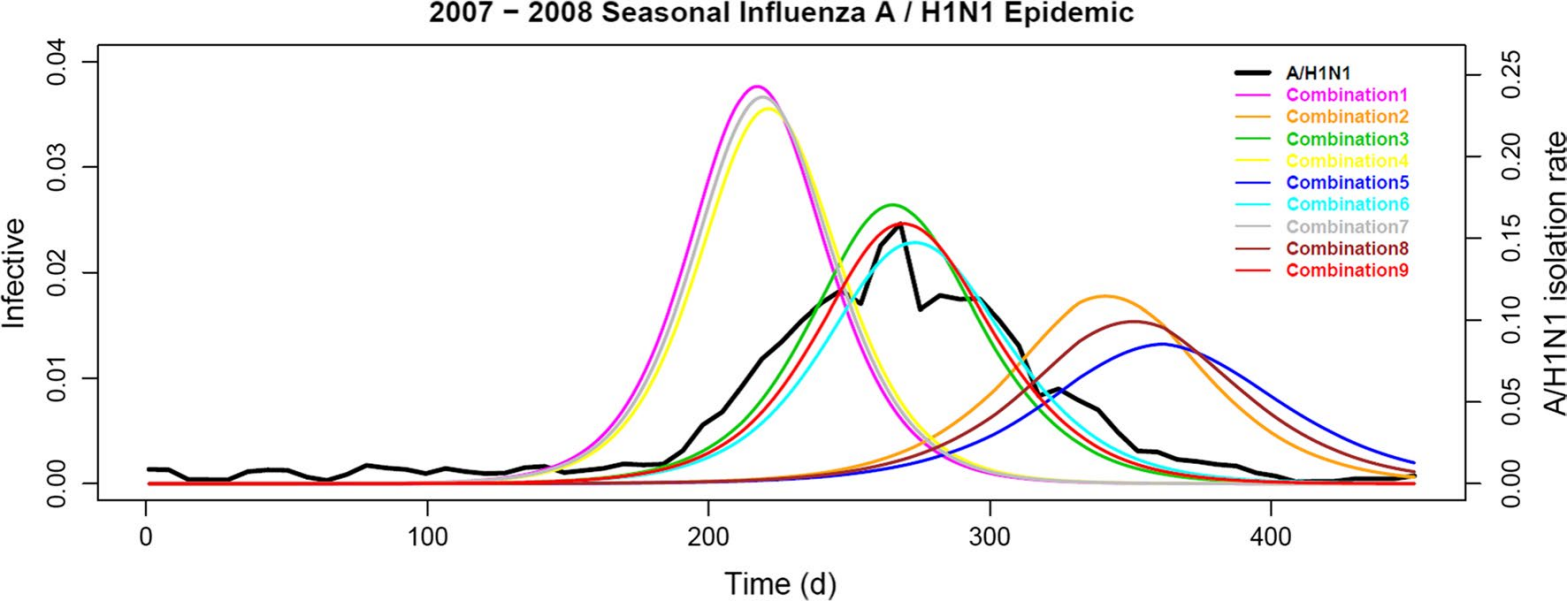
2007–2008 seasonal influenza A/H1N1 epidemic simulations. The model generated a simulated epidemic curve for each combination of *R*_0max_ and *R*_0min_. The combinations 1 through 9 presented in the figure, in the form of (*R*_0max_, *R*_0min_), were (1.65, 1.40), (1.65, 1.20), (1.65, 1.30), (1.45, 1.40), (1.45, 1.20), (1.45, 1.30), (1.55, 1.40), (1.55, 1.20), and (1.55, 1.30). The combination 9 simulated the observed weekly A/H1N1 IVIR curve (A/H1N1, shown in *black*) best and was 96.83% positively correlated. Against the time horizon, the proportion of the total population being infective was plotted on the *left vertical axis* and the weekly A/H1N1 IVIR on the *right*

To illustrate the effect of combinations of parameter values on the simulation curve and the sensitivity of the model to the parameter values, we selectively chose *R*_0max_, the maximum daily basic reproductive number [25], at 1.45, 1.55, and 1.65, and *R*_0min_, the minimum daily basic reproductive number [25], at 1.20, 1.30, and 1.40, and ran the model generating the epidemic curves for the nine combinations (Fig. 2). The weekly A/H1N1 IVIR curve, serving as the reference, was scaled by the best-fit simulation (set 1 in Table 2) for visual presentation. Obviously, higher *R*_0max_ or *R*_0min_ shifted the epidemic curve left with higher peak and lower *R*_0max_ or *R*_0min_ otherwise. Besides, *R*_0min_ dominated the main course of the epidemic curve and *R*_0max_ further shaped the simulation. All the epidemic curves converged to the best-fit simulation, and the ten best-fit that the model reported were shown earlier in Table 2.

**Table 2:** Parameter sets with corresponding characteristics of the ten best-fit simulations for the 2007–2008 influenza season

Set	Parameters		Characteristics			
	*R*_0max_^a^	*R*_0min_^a^	*I*_*X* peak_ (day)^b^	*I*_*X* max_ (%)^c^	Correlation coefficient (%)	*R*_e*X*_^d^
1	1.55	1.30	269	2.46	96.83	1.32
2	1.54	1.30	269	2.45	96.83	1.32
3	1.56	1.30	269	2.48	96.83	1.33
4	1.53	1.30	270	2.43	96.82	1.32
5	1.57	1.30	268	2.50	96.81	1.33
6	1.52	1.30	270	2.41	96.80	1.32
7	1.58	1.30	268	2.52	96.79	1.33
8	1.59	1.30	268	2.54	96.77	1.33
9	1.60	1.30	267	2.55	96.74	1.33
10	1.50	1.31	265	2.50	96.72	1.33
GM						1.33
GSD						1.00

341 simulations were performed with the parameters *R*_0max_ and *R*_0min_ set within each specified range. 10 best-fit simulations were selected based on the correlation coefficient between the simulated epidemic curve and the observed weekly A/H1N1 IVIR curve. Pearson method was used to estimate the correlation coefficient

*GM* geometric mean, *GSD* geometric standard deviation

^a^ *R*_0max_ and *R*_0min_ defined the range of the basic reproductive number [25]

^b^ *I*_*X*peak_ denoted the peak day of the epidemic curve which was manifested by the infectives of clade *X*

^c^ *I*_*X*max_ denoted the proportion of the total population being infectives of clade *X* on the peak day

^d^ *R*_e*X*_ estimated the annual effective reproductive number of clade *X*

### Fitting two-clade influenza season and estimating effective reproductive numbers

We continued to run the model under the context of the 2008–2009 influenza season and introduced clade *Y* on June 1 and clade *Z* on September 1, 2007. A successful simulation was defined by the following five criteria: (1) the correlation coefficient of at least 95% to the observed weekly A/H1N1 IVIR curve, (2) the peak of clade *Y* at most 15 days away from day 180, (3) the peak of clade *Z* at most 3 days away from the peak of the weekly A/H1N1 IVIR curve (day 304), (4) the height ratio of the peaks of clade *Z* to *Y* in the range between 192:10 and 192:7, and (5) the curve of clade *Y* crossing over the one of clade *Z* on the day at most 3 days away from day 210 (see “Methods” for details). However, the model reported that no successful simulation returned until strain fitness was taken into consideration. The ten best-fit simulations, ranked by the correlation coefficients, were shown in Table 3 (see Table 1 for details of the parameters). Through fitting the simulation to the weekly A/H1N1 IVIR curve as well as the clade-based A/H1N1 epidemiological curves, the model found not only successful simulations but also the potentially best one.

**Table 3:** Parameter sets with corresponding characteristics of the ten best-fit simulations for the 2008–2009 influenza season

Set	Parameters				Characteristics						
	*R*_0max_^a^	*R*_0min_^a^	*RF*_*Z*_^b^	*CC*_*Y*_^c^	*I*_*Y* peak_ (day)^d^	*I*_*Z* peak_ (day)^e^	*I*_*Z* max_/*I*_*Y* max_^f^	*I*_Y_*I_*Z*_ (day)^g^	Correlation coefficient (%)	*R*_e*Z*_^h^	*R*_e*Y*\*Z*_^i^
1	2.00	1.61	1.00	0.71	167	307	21.93	210	96.96	1.65	0.75
2	2.00	1.61	1.00	0.72	167	307	21.95	209	96.96	1.65	0.75
3	2.00	1.61	1.00	0.73	167	307	21.98	209	96.95	1.65	0.75
4	2.01	1.61	1.00	0.67	167	307	21.81	213	96.95	1.65	0.75
5	2.00	1.61	1.00	0.74	167	307	22.00	208	96.95	1.65	0.75
6	2.01	1.61	1.00	0.68	167	307	21.84	213	96.94	1.65	0.75
7	2.00	1.61	1.00	0.75	167	307	22.02	207	96.94	1.65	0.75
8	2.01	1.61	1.00	0.69	167	307	21.87	212	96.94	1.65	0.75
9	2.01	1.61	1.00	0.70	167	307	21.90	211	96.93	1.65	0.75
10	2.01	1.61	1.00	0.71	167	307	21.93	210	96.92	1.65	0.75
GM										1.65	0.75
GSD										1.00	1.00

13965 simulations were performed with the parameters *R*_0max_, *R*_0min_, *RF*_*Z*_, and *CC*_*Y*_ set within each specified range. 10 best-fit simulations were selected based on the correlation coefficient between the simulated epidemic curve and the observed weekly A/H1N1 IVIR curve. Pearson method was used to estimate the correlation coefficient

*GM* geometric mean, *GSD* geometric standard deviation

^a^ *R*_0max_ and *R*_0min_ defined the range of the basic reproductive number [25]

^b^ *RF*_*Z*_ represented relative fitness of clade *Z*

^c^ *CC*_*Y*_ represented competition cost of clade *Y*

^d^ *I*_*Y*peak_ denoted the peak day of clade *Y* epidemic which was manifested by the infectives of clade *Y*

^e^ *I*_*Z*peak_ denoted the peak day of clade *Z* epidemic which was manifested by the infectives of clade *Z*

^f^ *I*_*Zmax*_ */I*_*Ymax*_ denoted the height ratio of clade *Z* epidemic to clade *Y* epidemic

^g^ *I*_Y_*I_*Z*_ denoted the crossing day of clade *Z* epidemic with clade *Y* epidemic

^h^ *R*_e*Z*_ estimated the annual effective reproductive number of clade *Z*

^i^ *R*e*Y*\*Z* estimated the annual effective reproductive number of clade *Y* in the presence of clade *Z*

For the first parameter set, the model generated an epidemic curve that represented the summation for clades *Y* and *Z* and was 96.96% positively correlated to the observed weekly A/H1N1 IVIR curve. The curve of clade *Y* peaked on day 167 and declined thereafter. The curve of clade *Z* peaked on day 307 with a height 21.93 times that of clade *Y*. The two curves crossed on day 210. For clade *Z*, the monthly *R*_e_’s of the 15 months spanned by the simulation, corresponding to the months from April 2008 through June 2009, were estimated around 1.65, 1.64, 1.63, 1.62, 1.62, 1.63, 1.63, 1.66, 1.69, 1.70, 1.66, 1.67, 1.66, 1.65, and 1.63 with the annual *R*_e_ of around 1.65 (see Additional file 7: Table S6). As to clade *Y*, the annual *R*_e_ would originally be equivalent to 1.65 but finally took on around 0.75 after the introduction of clade *Z* (see Table 3 and Additional file  7: Table S6). The model reported that the strain fitness of clade *Z* was equivalent to that of clade *Y* (*RF*_*Z*_ = 1.00) and the latter encountered a reduction of strain fitness of around 71% (*CC*_*Y*_ = 0.71) while competing with the former. The model therefore could successfully identify the combination of *R*_0max_, *R*_0min_, *RF*_*Z*_ and *CC*_*Y*_ that best simulated the 2008–2009 influenza season and evaluate the *R*_e_’s of the co-circulating clades. In other words, in terms of the *R*_e_ estimated for each respective clade, the first parameter set along with the actual data embedded in the model could establish an epidemic 96.83% correlated to the real epidemic of 2008–2009 influenza season dominated by clade 2B-2 along with ever preceding clade 2C-2.

### Relative fitness declaring both clades per se equivalent in transmissibility

**Fig. 3:**
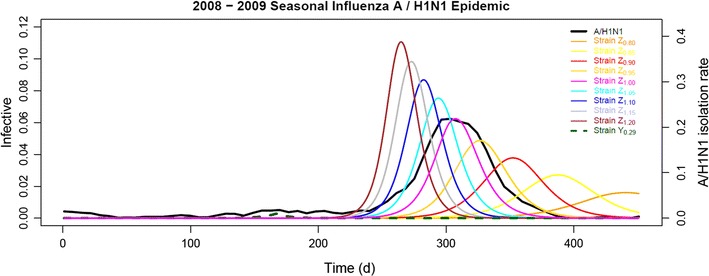
The effect of relative fitness of clade *Z* on 2008–2009 seasonal influenza A/H1N1 epidemic simulation. As clade *Y* assumed competition cost of 0.71 (Strain *Y*_0.29_), with *R*_0max_ and *R*_0min_ fixed at 2.00 and 1.61 respectively, the model generated epidemic curves of clade *Z* of a series of relative fitness from 0.80 (Strain *Z*_0.80_) through 1.20 (Strain *Z*_1.20_). Among them, the Strain *Z*_1.00_ simulated the observed weekly A/H1N1 IVIR curve (A/H1N1, shown in *black*) best and was 96.96% positively correlated. Against the time horizon, the proportion of the total population being infective was plotted on the *left vertical axis* and the weekly A/H1N1 IVIR on the *right*

To further illustrate the interaction between clades *Y* and *Z* and the sensitivity of the model to the *RF*_*Z*_ and *CC*_*Y*_ values, we would elaborate the simulation results in two ways. First, we selectively chose relative fitness of clade *Z* (*RF*_*Z*_) from 0.80 to 1.20 at intervals of 0.05 while fixing *R*_0max_ at 2.00, *R*_0min_ at 1.61, and competition cost of clade *Y* (*CC*_*Y*_) at 0.71, with the weekly A/H1N1 IVIR curve scaled by the best-fit simulation (set 1 in Table 3) as the reference. A spectrum of epidemic curves thereby generated by the model demonstrated the effect of *RF*_*Z*_ on the simulation curve (Fig. 3). We noticed that higher *RF*_*Z*_ shifted the simulated curve to the left with a higher peak and lower *RF*_*Z*_ to the right with the peak lower. In fact, strains of clade *Z* with relative fitness of 1.00 (Strain *Z*_1.00_) along with the preceding strains of clade *Y* suffering from competition cost of 0.71 (Strain *Y*_0.29_) initiated a simulated epidemic that correlated to the observed one represented by the weekly A/H1N1 IVIR curve best. All the epidemic curves converged to the best-fit simulation, and the ten best-fit that the model reported were shown earlier in Table 3.

### Competition cost explaining the dynamics between both clades

**Fig. 4:**
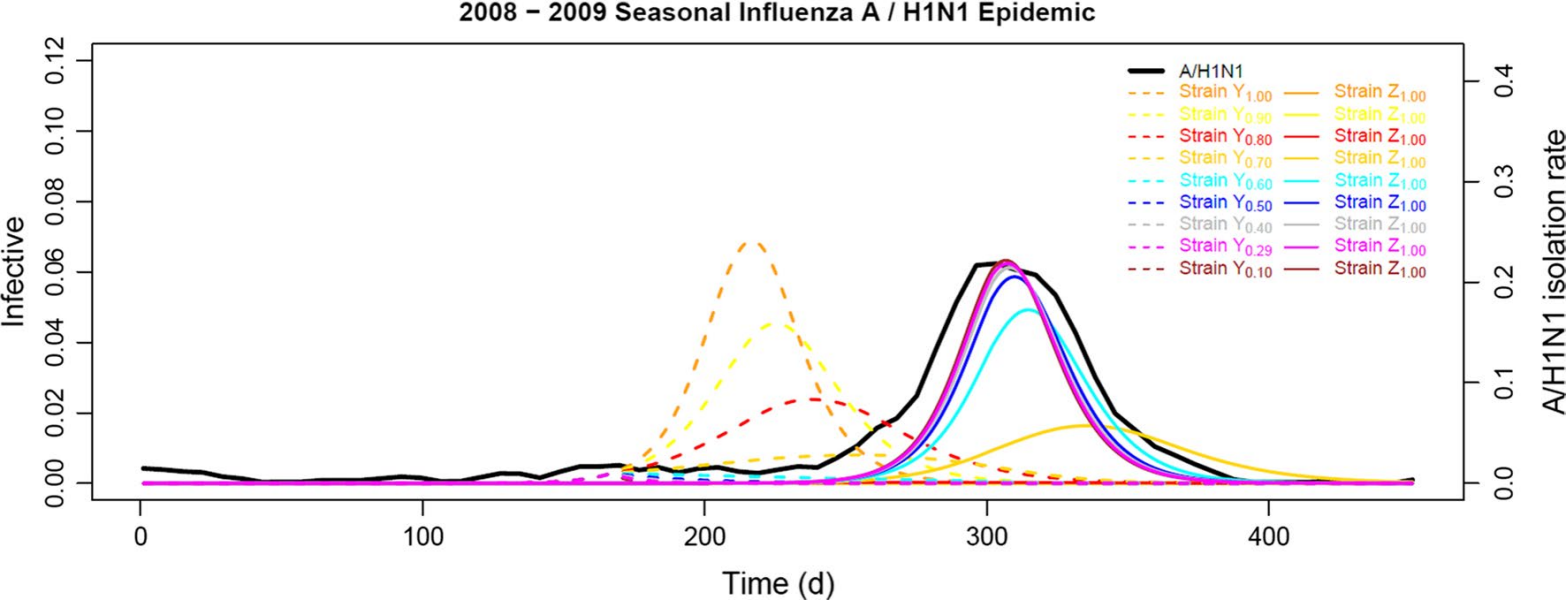
The effect of competition cost of clade *Y* on 2008–2009 seasonal influenza A/H1N1 epidemic simulation. With *R*_0max_ and *R*_0min_ fixed at 2.00 and 1.61 respectively, while clade *Z* assuming relative fitness of 1.00 (Strain *Z*_1.00_), the model generated the dynamic behavior of clade *Y* with competition cost between 0.00 (Strain *Y*_1.00_) and 0.90 (Strain *Y*_0.10_). The Strain *Y*_0.29_ coupled with Strain *Z*_1.00_ simulated the observed weekly A/H1N1 IVIR curve (A/H1N1, shown in *black*) best and was 96.96% positively correlated. Against the time horizon, the proportion of the total population being infective was plotted on the *left vertical axis* and the weekly A/H1N1 IVIR on the *right*

Secondly, we instead selectively chose *CC*_*Y*_ between 0.00 and 0.90 while fixing *R*_0max_ at 2.00, *R*_0min_ at 1.61, and *RF*_*Z*_ at 1.00, a rather intriguing pattern of the vicissitudes of two co-circulating clades was then unfolded (Fig. 4). Facing co-circulating clade *Z* equivalently fit to itself, if clade *Y* had not suffered much from the competition cost, i.e., if the *CC*_*Y*_ had been less than 0.20, it could have been the dominant clade during the 2008–2009 influenza season. However, once clade *Y* encountered a competition cost of more than 0.30, clade *Z* became the dominant one during the 2008–2009 influenza season. Moreover, strains of clade *Y* with competition cost of 0.71 (Strain *Y*_0.29_) coupled with the emerging strains of clade *Z* with relative fitness of 1.00 (Strain *Z*_1.00_) presented a simulated epidemic that correlated to the observed weekly A/H1N1 IVIR curve best. Again, all the epidemic curves converged to the best-fit simulation.

From the implementations stated above, the model reported Strain *Y*_0.29_ with Strain *Z*_1.00_ as the best combination of clades *Y* and *Z* co-circulating during the 2008–2009 influenza season. Through step-by-step exploration (see “Methods” for details), with *R*_0max_ set between 2.13 and 1.99, *R*_0min_ between 1.63 and 1.57, *RF*_*Z*_ between 1.03 and 0.97, and *CC*_*Y*_ between 0.60 and 0.78 in the final step, there were 617 successful simulations reported among the final 13,965 simulations. In the 617 successful simulations, we observed that *RF*_*Z*_ was in the range of 0.99 and 1.02 and *CC*_*Y*_ was in the range of 0.65 and 0.76. The findings strongly converged onto the scenario that the competition cost encountered by clade *Y* was involved in the interaction between the two clades.

## Discussion

On the basis of the results, we address three points pertinent to the 2008–2009 influenza season in Taiwan about strain competition in particular. First, in terms of *R*_e_, clade 2B-2 would be as fit as clade 2C-2, as evidenced by *RF*_*Z*_ of 1.00 derived from the transmission model. Although there were compelling evidences that the clinical effects of A/H1N1 H275Y variants, which circulated globally during the 2007–2009 influenza seasons, were consistent with normal seasonal activity [34, 35], the transmission efficiency of the variants, nevertheless, was not well studied but in one animal study [36]. In our study, the model not only reported that clade 2B-2, carrying H275Y mutation in the neuraminidase, transmitted as efficiently as clade 2C-2 but also revealed that the transmission efficiency of clade 2B-2 could not sufficiently explain the vicissitudes of the two co-circulating clades. The model demonstrated that clade 2B-2 outcompeted clade 2C-2 for the 2008–2009 influenza season mainly as a result of the competition cost that the latter incurred in the interaction between them.

Secondly, according to Hurt and Bloom et al. [37, 38], H275Y mutation was typically associated with poor replication and transmission, and permissive mutation could enable oseltamivir-resistant A/H1N1 variants to restore viral fitness. However, given that oseltamivir was rarely prescribed before May 2009 in Taiwan, in the absence of selection pressure from the antiviral, the replacement of clade 2C-2 with clade 2B-2 could not be well explained by the combination effect of H275Y mutation with permissive mutation. In our study, had there been no competition cost (refer to the Strain *Y*_1.00_ in Fig. 4), clade 2C-2 would have generated an earlier epidemic and, under the assumption of full cross-immunity, depleted compartment *S* to a level at which the real epidemic of clade 2B-2 would not have occurred. In terms of transmission dynamics, our model successfully adopted an ever innovative approach dissecting into strain interactions of influenza epidemics through a quantitative way. The embedded significance of the competition cost unveiled through our study probably plays an important role in deciphering the evolution trajectory of the influenza virus and deserves further research to discover its nature at the molecular level.

Thirdly, with higher-resolution information, integrating laboratory surveillance with modelling techniques could provide a good opportunity to simulate seasonal influenza epidemics specifically to the levels of type, clade, and even strain. Coherent with the findings of Shaman et al. [24, 25], absolute humidity could explain the theme of influenza seasonality at the population level in our study. However, to explore the competition of influenza strains, clade-based epidemiological data as well as weekly A/H1N1 IVIR were further exploited to excavate the underlying processes that might not be visible without skillful integration. The fact that our model could report for the 2008–2009 influenza season the best-fit simulations with correlation coefficient of up to 96% signifies the invaluable collaboration of, among others, laboratory, epidemiology, and mathematical modelling.

Through the transmission model we constructed, in terms of the *R*_e_’s estimated for clade 2B-2 and clade 2C-2, relative fitness (*RF*_*Z*_) and competition cost (*CC*_*Y*_) along with the basic reproductive number range (*R*_0max_ and *R*_0min_) could establish an epidemic 96.83% correlated to the real epidemic of 2008–2009 influenza season. Mathematical modelling has the potential to probe the seemingly intractable complexity of infectious disease dynamics and offers valuable tools to dissect epidemiological surveillance. As illustrated in the “Results” section (see Figs. 3, 4), the vicissitudes of two co-circulating clades could be elucidated and, in a sense, be predicted if we could further study the nature of relative fitness and competition cost. Besides, in our study, witnessing the emergence of the succeeding clade 2B-2 in September 2008 with the recession of the preceding clade 2C-2 on day 167 and even the displacement of the latter by the former on day 210 (see Table 3), our model would argue that it could provide a promising opportunity predicting the epidemic trend for an influenza season and assist in decision making for public health preparedness and response.

Facing the progress in the emergence of drug-resistant A/H1N1pdm09 variants, Oh et al. [39] proposed an experimental ferret model to assess the viral fitness of influenza viruses in terms of within-host replication and between-host transmission. Petrie et al. [40] further found that the within-host replication fitness of the oseltamivir-resistant A/H1N1pdm09 variants was not compromised relative to that of related oseltamivir-sensitive strains. Hurt et al. [15] analyzed a widespread community cluster of oseltamivir-resistant A/H1N1pdm09 influenza in Australia and warned that widespread emergence of oseltamivir-resistant A/H1N1pdm09 strains might be more likely. At the human population level, we were therefore concerned with the between-host transmission fitness of the drug-resistant A/H1N1pdm09 variants for better preparedness strategy and response capacity.

Being a study of the aggregate of individual persons, our study reflected the summation of the transmission dynamics of co-circulating influenza strains in the human population. Thanks to the contribution of virological surveillance, epidemiological documentation, vaccination program recording, demographic registry, and meteorological observations, the competition dynamics of co-circulating influenza strains could be explored in a quantitative way to simulate the real epidemic. Worthy of note, because our model took the advantage of the antiviral-free background before May 2009 in Taiwan, the results of our study were implicitly assumed independent of selection pressure exerted by antivirals and de novo antiviral resistance generated during prophylaxis or treatment. However, as sagacious antiviral usage becomes indispensable part of the portfolio for influenza control, factors concerning selection pressure, de novo resistance, and cross-resistance of antivirals shall be further incorporated into the transmission model in the case that the corresponding data could be available in the real word to facilitate the communication among basic, clinical, and population sciences.

Although clade-based epidemiological data were adopted, the scope of our study was actually at the human population level. Our model made an implied assumption that the interaction of influenza strains, through transmission dynamics, would manifest itself in the trend of seasonal epidemics. Because of the limited resolution of the laboratory surveillance in our study, we did not intend to investigate viral fitness at the molecular level, which is though fundamentally important. Nevertheless, we identified the nature and the magnitude of the interaction, and found that A/H1N1 clade 2B-2 outcompeted clade 2C-2 as the latter encountered the former and suffered from 71% reduction in the transmission fitness. Furthermore, we found that clade 2B-2 would be 24.06% higher in transmission fitness than clade 2B-1. These findings are not only in a sense consistent with the results from the animal model of Bouvier et al. [36] but also provide quantitative assessment of the enhanced human transmissibility of oseltamivir-resistant strains during the 2007–2009 influenza seasons.

## Conclusions

To our knowledge, this is the first time that the competition dynamics of co-circulating influenza strains was studied in a quantitative way at the human population level. Our study provides quantitative descriptions about the transmission efficiency of each circulating clade per se and brings to light the transmission dynamics of the A/H1N1 H275Y strains during the 2007–2009 influenza seasons worldwide. An interdisciplinary quantitative approach through mathematical modelling could probably facilitate the resolution process of the transmissibility of continually emerging drug-resistant A/H1N1pdm09 variants.

## Additional files

**Additional file 1: Table S1.** The weekly A/H1N1 influenza virus isolation rate (A/H1N1 IVIR) of the 2007-2008 and 2008-2009 influenza seasons. The surveillance system covered approximately 75% of the 352 basic administrative units throughout the northern, central, southern, and eastern regions of Taiwan. Considering that the original weekly data reflected the data variability of the national influenza surveillance network, we therefore adopted the aggregate weekly data, computed from all positive isolations divided by all collected specimens of the three consecutive weeks.

**Additional file 2: Table S2.** The mid-year population structure of the year 2007 and 2008. The mid-year age-structured population sizes were obtained from Department of Household Registration, Ministry of the Interior (http://www.ris.gov.tw/zh_TW/346). The model population was classified into six age groups: 0-5, 6-12, 13-19, 20-39, 40-59, and ≥ 60 years.

**Additional file 3: Appendix.** Transmission model construction description.

**Additional file 4: Table S3.** Meteorological data used to derive the monthly specific humidity during the 2007-2008 and 2008-2009 influenza seasons. Refer the meteorological variables used to derive specific humidity to Taiwan Central Weather Bureau (http://www.cwb.gov.tw/V7/climate/monthlyData/mD.htm).

**Additional file 5: Table S4.** Progress of annual influenza vaccination program from October 1 through December 30, year 2007 and 2008 respectively. The year 2007 influenza vaccination program started on October 1 and aimed at those aged 6 months through 2 years, the school children aged 6-7, and the elderly aged 65 years and above. From December 1 on, the program was open to the general population to maximize the vaccine utilization. As to the year 2008 influenza vaccination program, starting on October 1, it aimed at those aged 6 months through 3 years, the school children aged 6-9, and the elderly aged 65 years and above before December 1 and open to the general population thereafter

**Additional file 6: Table S5.** Parameter sets with the corresponding estimated monthly effective reproductive numbers and annual effective reproductive number of each simulation for the 2007-2008 influenza season. For a single-clade influenza season, the model found successful simulations and evaluated the effective reproductive numbers. The annual effective reproductive number was deduced from the geometric mean of the estimated monthly effective reproductive numbers. The range of the basic reproductive number was estimated accordingly.

**Additional file 7: Table S6.** Parameter sets with the corresponding estimated monthly effective reproductive numbers and annual effective reproductive number of each simulation for the 2008-2009 influenza season. For a two-clade influenza season, the model found successful simulations and evaluated the effective reproductive numbers. The annual effective reproductive number was deduced from the geometric mean of the estimated monthly effective reproductive numbers. Relative fitness of succeeding clade and competition cost of the preceding clade, as well as the range of the basic reproductive number, were estimated accordingly.

## Abbreviations

A/H1N1pdm09: pandemic (A/H1N1) 2009
CC: competition cost
IVIR: influenza virus isolation rate
*R*_e_: effective reproductive number
RF: relative fitness
