# Supplementary material for: Data-driven interdisciplinary mathematical modelling quantitatively unveils competition dynamics of co-circulating influenza strains
Source: J Transl Med. 2017 Jul 28;15:163. doi: 10.1186/s12967-017-1269-6 (PMC5534049; doi:10.1186/s12967-017-1269-6)
Supplement: Supplementary file 1 — Additional file 1: Table S1. The weekly A/H1N1 influenza virus isolation rate (A/H1N1 IVIR) of the 2007-2008 and 2008-2009 influenza seasons. The surveillance system covered approximately 75% of the 352 basic administrative units throughout the northern, central, southern, and eastern regions of Taiwan. Considering that the original weekly data reflected the data variability of the national influenza surveillance network, we therefore adopted the aggregate weekly data, computed from all positive isolations divided by all collected specimens of the three consecutive weeks. [file 12967_2017_1269_MOESM1_ESM.docx]

**Additional file 1: Table S1. The weekly A/H1N1 influenza virus isolation rate (A/H1N1 IVIR) of the 2007-2008 and 2008-2009 influenza seasons**

| Year / Week | Weekly data | Aggregate weekly data |
| --- | --- | --- |
| 2007 / 14 | 0.0180 | 0.0088 |
| 2007 / 15 | 0.0098 | 0.0085 |
| 2007 / 16 | 0.0000 | 0.0025 |
| 2007 / 17 | 0.0000 | 0.0025 |
| 2007 / 18 | 0.0089 | 0.0024 |
| 2007 / 19 | 0.0000 | 0.0074 |
| 2007 / 20 | 0.0135 | 0.0084 |
| 2007 / 21 | 0.0109 | 0.0082 |
| 2007 / 22 | 0.0000 | 0.0040 |
| 2007 / 23 | 0.0000 | 0.0020 |
| 2007 / 24 | 0.0055 | 0.0056 |
| 2007 / 25 | 0.0105 | 0.0112 |
| 2007 / 26 | 0.0159 | 0.0096 |
| 2007 / 27 | 0.0035 | 0.0087 |
| 2007 / 28 | 0.0074 | 0.0062 |
| 2007 / 29 | 0.0082 | 0.0093 |
| 2007 / 30 | 0.0126 | 0.0074 |
| 2007 / 31 | 0.0000 | 0.0062 |
| 2007 / 32 | 0.0047 | 0.0065 |
| 2007 / 33 | 0.0148 | 0.0098 |
| 2007 / 34 | 0.0103 | 0.0106 |
| 2007 / 35 | 0.0077 | 0.0068 |
| 2007 / 36 | 0.0036 | 0.0081 |
| 2007 / 37 | 0.0121 | 0.0094 |
| 2007 / 38 | 0.0123 | 0.0121 |
| 2007 / 39 | 0.0120 | 0.0115 |
| 2007 / 40 | 0.0103 | 0.0119 |
| 2007 / 41 | 0.0137 | 0.0202 |
| 2007 / 42 | 0.0330 | 0.0359 |
| 2007 / 43 | 0.0586 | 0.0439 |
| 2007 / 44 | 0.0435 | 0.0597 |
| 2007 / 45 | 0.0789 | 0.0763 |
| 2007 / 46 | 0.1003 | 0.0868 |
| 2007 / 47 | 0.0766 | 0.0995 |
| 2007 / 48 | 0.1256 | 0.1102 |
| 2007 / 49 | 0.1298 | 0.1180 |
| 2007 / 50 | 0.1034 | 0.1098 |
| 2007 / 51 | 0.1003 | 0.1453 |
| 2007 / 52 | 0.1937 | 0.1588 |
| 2008 / 1 | 0.1191 | 0.1064 |
| 2008 / 2 | 0.0958 | 0.1149 |
| 2008 / 3 | 0.1291 | 0.1126 |
| 2008 / 4 | 0.1117 | 0.1129 |
| 2008 / 5 | 0.0946 | 0.0999 |
| 2008 / 6 | 0.0645 | 0.0843 |
| 2008 / 7 | 0.0784 | 0.0537 |
| 2008 / 8 | 0.0347 | 0.0580 |
| 2008 / 9 | 0.0692 | 0.0511 |
| 2008 / 10 | 0.0511 | 0.0452 |
| 2008 / 11 | 0.0183 | 0.0315 |
| 2008 / 12 | 0.0258 | 0.0200 |
| 2008 / 13 | 0.0159 | 0.0194 |
| 2008 / 14 | 0.0157 | 0.0148 |
| 2008 / 15 | 0.0128 | 0.0136 |
| 2008 / 16 | 0.0123 | 0.0118 |
| 2008 / 17 | 0.0102 | 0.0108 |
| 2008 / 18 | 0.0102 | 0.0066 |
| 2008 / 19 | 0.0000 | 0.0046 |
| 2008 / 20 | 0.0039 | 0.0012 |
| 2008 / 21 | 0.0000 | 0.0014 |
| 2008 / 22 | 0.0000 | 0.0015 |
| 2008 / 23 | 0.0046 | 0.0030 |
| 2008 / 24 | 0.0044 | 0.0030 |
| 2008 / 25 | 0.0000 | 0.0031 |
| 2008 / 26 | 0.0048 | 0.0048 |
| 2008 / 27 | 0.0099 | 0.0065 |
| 2008 / 28 | 0.0048 | 0.0054 |
| 2008 / 29 | 0.0000 | 0.0019 |
| 2008 / 30 | 0.0000 | 0.0021 |
| 2008 / 31 | 0.0068 | 0.0060 |
| 2008 / 32 | 0.0118 | 0.0098 |
| 2008 / 33 | 0.0104 | 0.0095 |
| 2008 / 34 | 0.0060 | 0.0055 |
| 2008 / 35 | 0.0000 | 0.0115 |
| 2008 / 36 | 0.0294 | 0.0166 |
| 2008 / 37 | 0.0215 | 0.0167 |
| 2008 / 38 | 0.0000 | 0.0179 |
| 2008 / 39 | 0.0316 | 0.0132 |
| 2008 / 40 | 0.0063 | 0.0158 |
| 2008 / 41 | 0.0064 | 0.0107 |
| 2008 / 42 | 0.0196 | 0.0147 |
| 2008 / 43 | 0.0182 | 0.0158 |
| 2008 / 44 | 0.0119 | 0.0117 |
| 2008 / 45 | 0.0055 | 0.0101 |
| 2008 / 46 | 0.0124 | 0.0135 |
| 2008 / 47 | 0.0227 | 0.0167 |
| 2008 / 48 | 0.0148 | 0.0158 |
| 2008 / 49 | 0.0105 | 0.0256 |
| 2008 / 50 | 0.0474 | 0.0373 |
| 2008 / 51 | 0.0498 | 0.0551 |
| 2008 / 52 | 0.0598 | 0.0647 |
| 2009 / 1 | 0.0714 | 0.0870 |
| 2009 / 2 | 0.0971 | 0.1347 |
| 2009 / 3 | 0.2103 | 0.1800 |
| 2009 / 4 | 0.2258 | 0.2167 |
| 2009 / 5 | 0.1957 | 0.2185 |
| 2009 / 6 | 0.2140 | 0.2124 |
| 2009 / 7 | 0.2135 | 0.2072 |
| 2009 / 8 | 0.1969 | 0.1873 |
| 2009 / 9 | 0.1493 | 0.1499 |
| 2009 / 10 | 0.0836 | 0.1056 |
| 2009 / 11 | 0.0759 | 0.0695 |
| 2009 / 12 | 0.0503 | 0.0533 |
| 2009 / 13 | 0.0341 | 0.0366 |
| 2009 / 14 | 0.0235 | 0.0281 |
| 2009 / 15 | 0.0255 | 0.0192 |
| 2009 / 16 | 0.0084 | 0.0114 |
| 2009 / 17 | 0.0000 | 0.0038 |
| 2009 / 18 | 0.0031 | 0.0011 |
| 2009 / 19 | 0.0000 | 0.0011 |
| 2009 / 20 | 0.0000 | 0.0011 |
| 2009 / 21 | 0.0038 | 0.0013 |
| 2009 / 22 | 0.0000 | 0.0024 |
| 2009 / 23 | 0.0030 | 0.0012 |
| 2009 / 24 | 0.0000 | 0.0011 |
| 2009 / 25 | 0.0000 | 0.0000 |
| 2009 / 26 | 0.0000 | 0.0035 |

Note that the weekly data reflected the data variability of the national influenza surveillance network [[1](#_ENREF_1)]. The model therefore adopted the aggregate weekly data, computed from all positive isolations divided by all collected specimens of the three consecutive weeks.

**References**

1. Yang JR, Lin YC, Huang YP, Su CH, Lo J, Ho YL, Yao CY, Hsu LC, Wu HS, Liu MT. Reassortment and mutations associated with emergence and spread of oseltamivir-resistant seasonal influenza A/H1N1 viruses in 2005-2009. PLoS One. 2011;6(3):e18177.
