# Supplementary material for: Data-driven interdisciplinary mathematical modelling quantitatively unveils competition dynamics of co-circulating influenza strains
Source: J Transl Med. 2017 Jul 28;15:163. doi: 10.1186/s12967-017-1269-6 (PMC5534049; doi:10.1186/s12967-017-1269-6)
Supplement: Supplementary file 2 — Additional file 2: Table S2. The mid-year population structure of the year 2007 and 2008. The mid-year age-structured population sizes were obtained from Department of Household Registration, Ministry of the Interior (http://www.ris.gov.tw/zh_TW/346). The model population was classified into six age groups: 0-5, 6-12, 13-19, 20-39, 40-59, and ≥ 60 years. [file 12967_2017_1269_MOESM2_ESM.docx]

**Additional file 2: Table S2. The mid-year population structure of the year 2007 and 2008**

| Age group | Age | Persons |
| --- | --- | --- |
| year 2007 | | |
| 1 | 0-5 years | 1324591 |
| 2 | 6-12 years | 2116316 |
| 3 | 13-19 years | 2261730 |
| 4 | 20-39 years | 7425159 |
| 5 | 40-59 years | 6697130 |
| 6 | ≥ 60 years | 3092519 |
| Total 22917445 | | |
| year 2008 | | |
| 1 | 0-5 years | 1276430 |
| 2 | 6-12 years | 2044304 |
| 3 | 13-19 years | 2260330 |
| 4 | 20-39 years | 7390957 |
| 5 | 40-59 years | 6841666 |
| 6 | ≥ 60 years | 3184009 |
| Total 22997696 | | |

Source: Department of Household Registration, Ministry of the Interior (http://www.ris.gov.tw/zh_TW/346).
