# Supplementary material for: Data-driven interdisciplinary mathematical modelling quantitatively unveils competition dynamics of co-circulating influenza strains
Source: J Transl Med. 2017 Jul 28;15:163. doi: 10.1186/s12967-017-1269-6 (PMC5534049; doi:10.1186/s12967-017-1269-6)
Supplement: Supplementary file 3 — Additional file 3: Appendix. Transmission model construction description. [file 12967_2017_1269_MOESM3_ESM.docx]

**Additional file 3: Appendix. Transmission model construction description**

We intended to develop a transmission model with four dimensions taken into consideration, i.e., virological factors, epidemiological observations, social factors, and climatic factors [[1](#_ENREF_1)]. We constructed an *SEIR* (susceptible-exposed-infective-recovered) compartmental model as the backbone [[2](#_ENREF_2)]. Briefly, the model population was assumed completely susceptible to the emerging seasonal influenza at the outset with no discrimination among age groups. Influenza was introduced by assigning the initial infected person(s) within the susceptible compartment (*S*) on a specified day. People infected with influenza went through the exposed compartment (*E*) and then the infective compartment (*I*), and entered into the recovered compartment (*R*) to be immune. Individuals receiving influenza vaccination passed from compartment *S* directly into compartment *R*. The lengths of the latent and infectious periods were estimated averagely 1 day and 5 days, respectively [[3](#_ENREF_3)]. The equations below described the deterministic behaviors of the model (see Table 1 for details of the parameters). The size of compartment *I*, under the assumption that the infectives could reflect the influenza virus isolation rate, was set to track the virological data, the weekly A/H1N1 IVIR (see Supplementary Table 1), in pursuit of high correlation.

$$\frac{dS\left( t \right)}{dt}=-\lambda S\left( t \right) (1)$$

$$\frac{dE\left( t \right)}{dt}=\lambda S\left( t \right)-\theta E\left( t \right) (2)$$

$$\frac{dI\left( t \right)}{dt}=\theta E\left( t \right)-\alpha I\left( t \right) (3)$$

$$\frac{dR\left( t \right)}{dt}=\alpha I\left( t \right) (4)$$

Model fit was assessed by comparing the simulated epidemic curve, manifested by the size of compartment *I*, against the observed weekly A/H1N1 IVIR curve in both single-clade influenza season simulation and two-clade influenza season simulation. Pearson method was used to estimate the correlation coefficient. Owing to the limited resolution of the laboratory data regarding the seasonal influenza epidemic, we integrated the epidemiological evidence into the transmission model to further define the successful simulation. The criteria were thus defined correspondingly for single-clade epidemic simulation of the 2007-2008 influenza season and for two-clade epidemic simulation of the 2008-2009 influenza season (see the Results section for details).

We continued to incorporate the social factors into the transmission model in the following way. The age structure was incorporated into the model population as the first step and the normalized age-specific contact rate, derived from social contact data [[4](#_ENREF_4)], was then applied to the pertinent age group. Age-related transmission rates were calculated based on the normalized age-specific contact rates and the spectral radius of the next generation matrix for heterogeneous population [[5](#_ENREF_5)]. The transmission rate coefficient for age group *i* from age group *j*, *β*[*i*, *j*], was hence given by

$$\beta\left[ i,j \right]=\frac{R_{e}\times cr\left[ i,j \right]}{\rho\left( K \right)}\times\frac{\alpha+\mu}{N_{t}}\cong\frac{R_{e}\times cr\left[ i,j \right]}{\rho\left( K \right)}\times\frac{\alpha}{N_{t}} (5)$$

where *cr*[*i*, *j*], *ρ*(*K*), *α, μ*, and *N_t_* denoted the contact rate of age group *i* by age group *j*, the spectral radius of the next generation matrix for heterogeneous population, the recovery rate, the death rate, and the total population size, respectively [[5](#_ENREF_5)]. As the time scale of life, 1/*μ*, being much larger than the time scale of the infectious period, 1/*α*, the death rate was ignored. The next generation matrix *K* was defined by

$$K\left[ i,j \right]=cr\left[ j,i \right]\times\frac{N_{i}}{N_{t}} (6)$$

where *N_i_* denoted the population size of age group *i*. As a result, the force of infection to age group *i*, *λ*[*i*], followed the expression

$$\lambda\left[ i \right]=\sum_{j=\text{1}}^{\text{6}} \beta\left[ i,j \right]\times I\left[ j \right] (7)$$

where *I*[*j*] represented the size of age group *j* in compartment *I*. As the time scale of life, 1/*μ*, being much larger than the time scale of the infectious period, 1/*α*, the death rate could be ignored. For simplicity, we assumed that influenza was introduced into the age-structured population by assigning one infected person in each age group within compartment *S* to spark off the initial spread of influenza.

With regard to the climatic factors, we explored in the transmission model the seasonality that shaped the epidemic curve all through an influenza season. On probing into the possibility that absolute humidity could drive seasonal variations of influenza transmission [[6](#_ENREF_6), [7](#_ENREF_7)], we demonstrated its utilization in the single-clade epidemic simulation of the 2007-2008 influenza season, as presented in the Results section. The simulations were performed step by step with the parameters *R*_0max_ set ranging from 2.40 to 1.20 and *R*_0min_ set ranging from *R*_0max_ to 0.80, at intervals of 0.1, 0.05, and 0.01 successively with the best-fit picked up as the reference point for the next step. We then took up this approach as the basis on which the two-clade epidemic simulation of the 2008-2009 influenza season was performed.

However, viewing the finding that it would be impossible to reproduce the scenario of the 2008-2009 influenza season unless the preceding clade incurred reduction in transmissibility on encountering the succeeding clade, the model further tried probing into strain fitness in two ways. On one hand, since we were interested in whether the succeeding clade per se had fitness advantage over the preceding one, we defined relative fitness (*RF*) to quantify the ratio of transmissibility, in terms of *R*_e_, of the former over the latter as the expression below.

*RF* = *R*_e_ _succeeding clade_ / *R*_e_ _preceding clade_ (8)

On the other hand, because we were also interested in whether the preceding clade suffered from transmissibility impairment when encountering the succeeding one, we defined competition cost (*CC*) to quantify the possible reduction in transmissibility of the former associated with the interaction between the two clades as the expresion below.

*CC* = 1 – ( *R*_e_ _preceding clade\succeeding clade_ / *R*_e_ _preceding clade_ ) (9)

The simulations were performed step by step with the parameters *R*_0max_ set ranging from 2.40 to 1.20, *R*_0min_ ranging from *R*_0max_ to 0.80, *CC* ranging from 0.30 to 0.90, and *RF* ranging from 0.90 to 1.10, at intervals of 0.1, 0.05, and 0.01 successively with the best-fit picked up as the reference point for the next step.

To simulate a single-clade influenza season, clade *X*, being oseltamivir-sensitive, represented the strains circulating in the 2007-2008 influenza season in Taiwan. In line with the scenario of the 2008-2009 influenza season in Taiwan, two more clades of A/H1N1 virus were defined: clade *Y* being oseltamivir-sensitive and clade *Z* being oseltamivir-resistant. All three clades were predominantly reported as low reactors to the contemporaneous annual vaccine strains [[8](#_ENREF_8)]. We therefore assumed 50% of the vaccinees were protected from infection of the contemporaneous seasonal strains [[9](#_ENREF_9), [10](#_ENREF_10)] and passed from compartment *S* directly into compartment *R* 15 days after vaccination [[11](#_ENREF_11)]. For simplicity, full cross-immunity was further assumed for clades *Y* and *Z*, and people infected by either clade entered into compartment *R* without differentiation. The simulation spanned 15 months, covering the length of an annual influenza season and extending 3 months forward so that the observed emerging stage of the seasonal influenza epidemic could be included (see Materials and Methods for details).

**References**

1. World Health Organization. Pandemic Influenza Risk Management. WHO Interim Guidance. World Health Organization, Geneva, 2013.

2. Anderson RM, May RM. Infectious Diseases of Humans: Dynamics and Control. Oxford Univ. Press, Oxford; 1991.

3. Carrat F, Vergu E, Ferguson NM, Lemaitre M, Cauchemez S, Leach S, Valleron AJ. Time lines of infection and disease in human influenza: a review of volunteer challenge studies. Am J Epidemiol. 2008;167(7):775-785.

4. Wallinga J, Teunis P, Kretzschmar M. Using data on social contacts to estimate age-specific transmission parameters for respiratory-spread infectious agents. Am J Epidemiol. 2006;164(10):936-944.

5. Diekmann O, Heesterbeek JAP. Mathematical Epidemiology of Infectious Diseases: Model Building, Analysis and Interpretation. John Wiley & Sons, Ltd, Chichester; 2000.

6. Shaman J, Pitzer VE, Viboud C, Grenfell BT, Lipsitch M. Absolute humidity and the seasonal onset of influenza in the continental United States. PLoS Biol. 2010;8(2):e1000316.

7. Shaman J, Kohn M. Absolute humidity modulates influenza survival, transmission, and seasonality. Proc Natl Acad Sci. 2009;106(9):3243-3248.

8. Levy A, Sullivan SG, Tempone SS, Wong KL, Regan AK, Dowse GK, Effler PV, Smith DW. Influenza vaccine effectiveness estimates for Western Australia during a period of vaccine and virus strain stability, 2010 to 2012. Vaccine. 2014;32(47):6312-6318.

9. Hannoun C, Megas F, Piercy J. Immunogenicity and protective efficacy of influenza vaccination. Virus Res. 2004;103(1-2):133-138.

10. Clements ML, Betts RF, Tierney EL, Murphy BR. Serum and nasal wash antibodies associated with resistance to experimental challenge with influenza A wild-type virus. J Clin Microbiol. 1986;24:157-160.

11. Moldoveanu Z, Clements ML, Prince SJ, Murphy BR, Mestecky J. Human immune responses to influenza virus vaccines administered by systemic or mucosal routes. Vaccine. 1995;13:1006-1012.
