# Supplementary material for: Data-driven interdisciplinary mathematical modelling quantitatively unveils competition dynamics of co-circulating influenza strains
Source: J Transl Med. 2017 Jul 28;15:163. doi: 10.1186/s12967-017-1269-6 (PMC5534049; doi:10.1186/s12967-017-1269-6)
Supplement: Supplementary file 4 — Additional file 4: Table S3. Meteorological data used to derive the monthly specific humidity during the 2007-2008 and 2008-2009 influenza seasons. Refer the meteorological variables used to derive specific humidity to Taiwan Central Weather Bureau (http://www.cwb.gov.tw/V7/climate/monthlyData/mD.htm). [file 12967_2017_1269_MOESM4_ESM.docx]

**Additional file 4: Table S3. Meteorological data used to derive the monthly specific humidity during the 2007-2008 and 2008-2009 influenza seasons**

| Meteorological variables | Influenza season | Apr | May | Jun | Jul | Aug | Sep | Oct | Nov | Dec | Jan | Feb | Mar | Apr | May | Jun |
| --- | --- | --- | --- | --- | --- | --- | --- | --- | --- | --- | --- | --- | --- | --- | --- | --- |
| *Temp* (°C) | 2007-2008  2008-2009 | 21.2  22.5 | 26.4  24.9 | 28.0  27.5 | 30.4  29.6 | 28.7  29.7 | 27.5  27.8 | 24.8  25.9 | 20.7  21.6 | 19.3  18.4 | 16.5  15.7 | 14.1  20.2 | 19.3  18.6 | 22.5  21.3 | 24.9  25.3 | 27.5  28.2 |
| *RH* (%) | 2007-2008  2008-2009 | 75  77 | 71  73 | 80  78 | 69  72 | 76  70 | 79  76 | 76  76 | 78  74 | 74  70 | 81  74 | 83  77 | 71  78 | 77  72 | 73  65 | 78  73 |
| *AP* (hPa) | 2007-2008  2008-2009 | 1014.0  1012.5 | 1008.9  1008.1 | 1006.0  1006.9 | 1005.8  1004.6 | 1003.8  1006.1 | 1006.3  1005.9 | 1012.6  1014.2 | 1017.3  1018.2 | 1018.3  1020.2 | 1020.3  1022.1 | 1021.3  1015.6 | 1016.1  1015.9 | 1012.5  1013.4 | 1008.1  1010.9 | 1006.9  1005.0 |

Note that *Temp*, *RH*, and *AP* denoted mean temperature, mean relative humidity, and mean atmospheric pressure, respectively, at Taipei. The final specific humidity unit was in gm/kg.
