# Supplementary material for: Data-driven interdisciplinary mathematical modelling quantitatively unveils competition dynamics of co-circulating influenza strains
Source: J Transl Med. 2017 Jul 28;15:163. doi: 10.1186/s12967-017-1269-6 (PMC5534049; doi:10.1186/s12967-017-1269-6)
Supplement: Supplementary file 5 — Additional file 5: Table S4. Progress of annual influenza vaccination program from October 1 through December 30, year 2007 and 2008 respectively. The year 2007 influenza vaccination program started on October 1 and aimed at those aged 6 months through 2 years, the school children aged 6-7, and the elderly aged 65 years and above. From December 1 on, the program was open to the general population to maximize the vaccine utilization. As to the year 2008 influenza vaccination program, starting on October 1, it aimed at those aged 6 months through 3 years, the school children aged 6-9, and the elderly aged 65 years and above before December 1 and open to the general population thereafter [file 12967_2017_1269_MOESM5_ESM.docx]

**Additional file 5: Table S4. Progress of annual influenza vaccination program from October 1 through December 30, year 2007 and 2008 respectively**

| Period | Persons vaccinated in age group | | | |
| --- | --- | --- | --- | --- |
|  | 0-5 years | 6-12 years | 13-59 years | ≥ 60 years |
| year 2007 | | | | |
| October 1-14 | 15332 | 7778 | 0 | 598216 |
| October 15-31 | 17871 | 14745 | 0 | 374817 |
| November 1-15 | 24993 | 66058 | 0 | 86739 |
| November 16-30 | 24518 | 199748 | 0 | 32898 |
| December 1-15 | 40513 | 79129 | 176721 | 41485 |
| December 16-30 | 20829 | 20135 | 92864 | 24209 |
| year 2008 | | | | |
| October 1-14 | 39291 | 94894 | 0 | 694292 |
| October 15-31 | 37722 | 375137 | 0 | 374733 |
| November 1-15 | 39933 | 210918 | 0 | 96990 |
| November 16-30 | 40701 | 125405 | 0 | 61199 |
| December 1-15 | 43758 | 46253 | 329851 | 64072 |
| December 16-30 | 9915 | 1577 | 4568 | 21182 |

Note that the Period column was specified to accommodate to data availability and the age groups were adjusted in accordance with those adopted in the model. The year 2008 influenza vaccination program started on October 1 and aimed at those aged 6 months through 3 years, the school children aged 6-9, and the elderly aged 65 years and above. From December 1 on, the program was open to the general population to maximize the vaccine utilization. As to the year 2007 influenza vaccination program, it was aimed at those aged 6 months through 2 years, the school children aged 6-7, and the elderly aged 65 years and above before December 1 and open to the general population thereafter.
