# Supplementary material for: Data-driven interdisciplinary mathematical modelling quantitatively unveils competition dynamics of co-circulating influenza strains
Source: J Transl Med. 2017 Jul 28;15:163. doi: 10.1186/s12967-017-1269-6 (PMC5534049; doi:10.1186/s12967-017-1269-6)
Supplement: Supplementary file 6 — Additional file 6: Table S5. Parameter sets with the corresponding estimated monthly effective reproductive numbers and annual effective reproductive number of each simulation for the 2007-2008 influenza season. For a single-clade influenza season, the model found successful simulations and evaluated the effective reproductive numbers. The annual effective reproductive number was deduced from the geometric mean of the estimated monthly effective reproductive numbers. The range of the basic reproductive number was estimated accordingly. [file 12967_2017_1269_MOESM6_ESM.docx]

**Additional file 6: Table S5. Parameter sets with the corresponding estimated monthly effective reproductive numbers and annual effective reproductive number of each simulation for the 2007-2008 influenza season**

| Set | Parameters | | Monthly effective reproductive number | | | | | | | | | | | | | | | *R*_e_*_X_* |
| --- | --- | --- | --- | --- | --- | --- | --- | --- | --- | --- | --- | --- | --- | --- | --- | --- | --- | --- |
|  | *R*_0max_ | *R*_0min_ | Apr  2007 | May  2007 | Jun  2007 | Jul  2007 | Aug  2007 | Sep  2007 | Oct  2007 | Nov  2007 | Dec  2007 | Jan  2008 | Feb  2008 | Mar  2008 | Apr  2008 | May  2008 | Jun  2008 |  |
| 1 | 1.55 | 1.30 | 1.33 | 1.32 | 1.31 | 1.31 | 1.31 | 1.31 | 1.32 | 1.33 | 1.34 | 1.35 | 1.36 | 1.34 | 1.32 | 1.32 | 1.31 | 1.32 |
| 2 | 1.54 | 1.30 | 1.33 | 1.32 | 1.31 | 1.31 | 1.31 | 1.31 | 1.32 | 1.33 | 1.34 | 1.34 | 1.36 | 1.34 | 1.32 | 1.32 | 1.31 | 1.32 |
| 3 | 1.56 | 1.30 | 1.33 | 1.32 | 1.31 | 1.31 | 1.31 | 1.31 | 1.32 | 1.33 | 1.34 | 1.35 | 1.36 | 1.34 | 1.33 | 1.32 | 1.31 | 1.33 |
| 4 | 1.53 | 1.30 | 1.33 | 1.31 | 1.31 | 1.31 | 1.31 | 1.31 | 1.32 | 1.33 | 1.34 | 1.34 | 1.35 | 1.34 | 1.32 | 1.32 | 1.31 | 1.32 |
| 5 | 1.57 | 1.30 | 1.33 | 1.32 | 1.31 | 1.31 | 1.31 | 1.31 | 1.32 | 1.33 | 1.34 | 1.35 | 1.36 | 1.35 | 1.33 | 1.32 | 1.31 | 1.33 |
| 6 | 1.52 | 1.30 | 1.33 | 1.31 | 1.31 | 1.31 | 1.31 | 1.31 | 1.32 | 1.33 | 1.34 | 1.34 | 1.35 | 1.34 | 1.32 | 1.32 | 1.31 | 1.32 |
| 7 | 1.58 | 1.30 | 1.33 | 1.32 | 1.31 | 1.31 | 1.31 | 1.31 | 1.32 | 1.33 | 1.34 | 1.35 | 1.36 | 1.35 | 1.33 | 1.32 | 1.31 | 1.33 |
| 8 | 1.59 | 1.30 | 1.34 | 1.32 | 1.31 | 1.31 | 1.31 | 1.31 | 1.32 | 1.34 | 1.35 | 1.35 | 1.37 | 1.35 | 1.33 | 1.32 | 1.31 | 1.33 |
| 9 | 1.60 | 1.30 | 1.34 | 1.32 | 1.31 | 1.31 | 1.31 | 1.31 | 1.32 | 1.34 | 1.35 | 1.36 | 1.37 | 1.35 | 1.33 | 1.32 | 1.31 | 1.33 |
| 10 | 1.50 | 1.31 | 1.33 | 1.32 | 1.32 | 1.32 | 1.32 | 1.32 | 1.32 | 1.33 | 1.34 | 1.35 | 1.35 | 1.34 | 1.33 | 1.32 | 1.32 | 1.33 |

Note that *R*_e_*_X_* was defined as the geometric mean of the estimated monthly effective reproductive numbers of the same set and represented the estimated annual effective reproductive number of clade *X* for the 2007-2008 influenza season. *R*_0max_ and *R*_0min_ defined the range of the basic reproductive number [[1](#_ENREF_1)].

**References**

1. Shaman J, Pitzer VE, Viboud C, Grenfell BT, Lipsitch M. Absolute humidity and the seasonal onset of influenza in the continental United States. PLoS Biol. 2010;8(2):e1000316.
