# Supplementary material for: Data-driven interdisciplinary mathematical modelling quantitatively unveils competition dynamics of co-circulating influenza strains
Source: J Transl Med. 2017 Jul 28;15:163. doi: 10.1186/s12967-017-1269-6 (PMC5534049; doi:10.1186/s12967-017-1269-6)
Supplement: Supplementary file 7 — Additional file 7: Table S6. Parameter sets with the corresponding estimated monthly effective reproductive numbers and annual effective reproductive number of each simulation for the 2008-2009 influenza season. For a two-clade influenza season, the model found successful simulations and evaluated the effective reproductive numbers. The annual effective reproductive number was deduced from the geometric mean of the estimated monthly effective reproductive numbers. Relative fitness of succeeding clade and competition cost of the preceding clade, as well as the range of the basic reproductive number, were estimated accordingly. [file 12967_2017_1269_MOESM7_ESM.docx]

**Additional file 7: Table S6. Parameter sets with the corresponding estimated monthly effective reproductive numbers and annual effective reproductive number of each simulation for the 2008-2009 influenza season**

| Set | Parameters | | | | Monthly effective reproductive number of clade *Z* | | | | | | | | | | | | | | | *R*_e_*_Z_* | *R*_e_*_Y_*_\_*_Z_* |
| --- | --- | --- | --- | --- | --- | --- | --- | --- | --- | --- | --- | --- | --- | --- | --- | --- | --- | --- | --- | --- | --- |
|  | *R*_0max_ | *R*_0min_ | *RF_Z_* | *CC_Y_* | Apr  2008 | May  2008 | Jun  2008 | Jul  2008 | Aug  2008 | Sep  2008 | Oct  2008 | Nov  2008 | Dec  2008 | Jan  2009 | Feb  2009 | Mar  2009 | Apr  2009 | May  2009 | Jun  2009 |  |  |
| 1 | 2.00 | 1.61 | 1.00 | 0.71 | 1.65 | 1.64 | 1.63 | 1.62 | 1.62 | 1.63 | 1.63 | 1.66 | 1.69 | 1.70 | 1.66 | 1.67 | 1.66 | 1.65 | 1.63 | 1.65 | 0.75 |
| 2 | 2.00 | 1.61 | 1.00 | 0.72 | 1.65 | 1.64 | 1.63 | 1.62 | 1.62 | 1.63 | 1.63 | 1.66 | 1.69 | 1.70 | 1.66 | 1.67 | 1.66 | 1.65 | 1.63 | 1.65 | 0.75 |
| 3 | 2.00 | 1.61 | 1.00 | 0.73 | 1.65 | 1.64 | 1.63 | 1.62 | 1.62 | 1.63 | 1.63 | 1.66 | 1.69 | 1.70 | 1.66 | 1.67 | 1.66 | 1.65 | 1.63 | 1.65 | 0.75 |
| 4 | 2.01 | 1.61 | 1.00 | 0.67 | 1.65 | 1.64 | 1.63 | 1.62 | 1.62 | 1.63 | 1.63 | 1.66 | 1.69 | 1.70 | 1.66 | 1.67 | 1.66 | 1.65 | 1.63 | 1.65 | 0.75 |
| 5 | 2.00 | 1.61 | 1.00 | 0.74 | 1.65 | 1.64 | 1.63 | 1.62 | 1.62 | 1.63 | 1.63 | 1.66 | 1.69 | 1.70 | 1.66 | 1.67 | 1.66 | 1.65 | 1.63 | 1.65 | 0.75 |
| 6 | 2.01 | 1.61 | 1.00 | 0.68 | 1.65 | 1.64 | 1.63 | 1.62 | 1.62 | 1.63 | 1.63 | 1.66 | 1.69 | 1.70 | 1.66 | 1.67 | 1.66 | 1.65 | 1.63 | 1.65 | 0.75 |
| 7 | 2.00 | 1.61 | 1.00 | 0.75 | 1.65 | 1.64 | 1.63 | 1.62 | 1.62 | 1.63 | 1.63 | 1.66 | 1.69 | 1.70 | 1.66 | 1.67 | 1.66 | 1.65 | 1.63 | 1.65 | 0.75 |
| 8 | 2.01 | 1.61 | 1.00 | 0.69 | 1.65 | 1.64 | 1.63 | 1.62 | 1.62 | 1.63 | 1.63 | 1.66 | 1.69 | 1.70 | 1.66 | 1.67 | 1.66 | 1.65 | 1.63 | 1.65 | 0.75 |
| 9 | 2.01 | 1.61 | 1.00 | 0.70 | 1.65 | 1.64 | 1.63 | 1.62 | 1.62 | 1.63 | 1.63 | 1.66 | 1.69 | 1.70 | 1.66 | 1.67 | 1.66 | 1.65 | 1.63 | 1.65 | 0.75 |
| 10 | 2.01 | 1.61 | 1.00 | 0.71 | 1.65 | 1.64 | 1.63 | 1.62 | 1.62 | 1.63 | 1.63 | 1.66 | 1.69 | 1.70 | 1.66 | 1.67 | 1.66 | 1.65 | 1.63 | 1.65 | 0.75 |

Note that *R*_eZ_ was defined as the geometric mean of the estimated monthly effective reproductive numbers of clade Z and represented its estimated annual effective reproductive number for the 2008-2009 influenza season. Defined as a geometric mean of the estimated monthly effective reproductive numbers of clade *Y* in the presence of clade *Z*, *R*_e_*_Y_*_\_*_Z_* denoted the estimated annual conditional effective reproductive number of clade *Z* for the 2008-2009 influenza season. *R*_0max_ and *R*_0min_ defined the range of the basic reproductive number [[1](#_ENREF_1)]. *RF_Z_* represented relative fitness of clade *Z*, and *CC_Y_*, competition cost of clade *Y.*

**References**

1. Shaman J, Pitzer VE, Viboud C, Grenfell BT, Lipsitch M. Absolute humidity and the seasonal onset of influenza in the continental United States. PLoS Biol. 2010;8(2):e1000316.
